# Cognitive Components of Regularity Processing in the Auditory Domain

**DOI:** 10.1371/journal.pone.0002650

**Published:** 2008-07-09

**Authors:** Stefan Koelsch, Daniela Sammler

**Affiliations:** 1 University of Sussex, Department of Psychology, Brighton, United Kingdom; 2 Max Planck Institute for Human Cognitive and Brain Science, Leipzig, Germany; James Cook University, Australia

## Abstract

**Background:**

Music-syntactic irregularities often co-occur with the processing of physical irregularities. In this study we constructed chord-sequences such that perceived differences in the cognitive processing between regular and irregular chords could not be due to the sensory processing of acoustic factors like pitch repetition or pitch commonality (the major component of ‘sensory dissonance’).

**Methodology/Principal Findings:**

Two groups of subjects (musicians and nonmusicians) were investigated with electroencephalography (EEG). Irregular chords elicited an early right anterior negativity (ERAN) in the event-related brain potentials (ERPs). The ERAN had a latency of around 180 ms after the onset of the music-syntactically irregular chords, and had maximum amplitude values over right anterior electrode sites.

**Conclusions/Significance:**

Because irregular chords were hardly detectable based on acoustical factors (such as pitch repetition and sensory dissonance), this ERAN effect reflects for the most part cognitive (not sensory) components of regularity-based, music-syntactic processing. Our study represents a methodological advance compared to previous ERP-studies investigating the neural processing of music-syntactically irregular chords.

## Introduction

**Figure 1:**
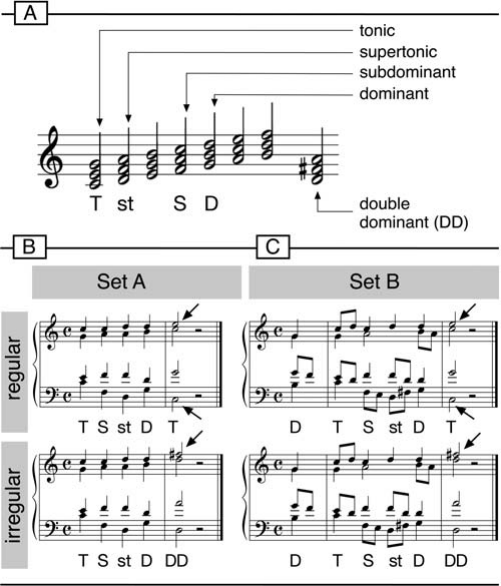
A: Illustration of chord functions. The chord built on the first scale tone is denoted as the tonic, the chord on the second scale tone as the supertonic, on the fourth scale tone as subdominant, and on the fifth scale tone as the dominant. The major chord on the second tone of a scale can be interpreted as the dominant to the dominant, or double dominant (DD). B: Examples of stimulus Set A (“homophonic”). Chord sequences ended either on a tonic chord (T, regular), or on a double dominant (DD, irregular). Arrows indicate pitches that were not contained in the preceding chords. C: Examples of stimulus Set B (“polyphonic”). As in Set A, chord sequences ended either on a tonic chord (T, regular), or on a double dominant (DD, irregular). Arrows indicate pitches that were not contained in the preceding chords. In the experiment, sequences from all twelve major keys were presented in direct succession, the tonal key changed from sequence to sequence, and both regular and irregular sequence endings occurred randomly with equal probability (0.5). Stimulus sets were presented in blocks, counterbalanced across subjects.

Since the mid 1980s, a number of studies from different groups investigated the neural mechanisms underlying the processing of musical structure using event-related brain potentials (ERPs). Such investigations were not only driven by the interest in brain mechanisms underlying the processing of music, but also by the question of how the brain extracts, memorizes, and applies knowledge about regularities underlying sequential auditory information in a domain other than language e.g., [1]–[5]. So far, the most fruitful approach has been to present participants with sequences of chords and compare electric brain responses to regular chord functions with those to harmonically irregular chord functions (see Figure 1A for explanation of the term “chord function”). For example, Figure 1B shows two chord sequences of which chords 1 to 4 are arranged in a fashion that, according to the theory of harmony, the most regular chord function at the final (fifth) position is the tonic (e.g. [6]–[7], see upper panel of Figure 1B). Previous studies have used experimental stimuli in which the tonic at the final position was replaced by harmonically less regular chords, such as a “*Neapolitan sixth chord*” e.g. [8]–[10], a *supertonic* [11], or a *double dominant* [11] (DD; the DD is the major chord built on the second scale tone, see also lower panel of Figure 1B; a double dominant [in major] is often also referred to as *chromatic supertonic*.).

The regularities of the arrangement of chord functions within a harmonic sequence have been denoted as part of a musical syntax [12], [13], [3], and previous studies examining neural mechanisms of processing musical syntax using chord sequence paradigms revealed a variety of ERP components to be elicited by irregular harmonies, such as the P300 [14], LPC (late positive component, [15]), RATN (right anterior temporal negativity, [1]), and ERAN (early right anterior negativity, [8]); the functional significance of these components has been reviewed elsewhere [16], [17], [18].

As we have already pointed out previously [11], investigations on the processing of musical structure using chord sequence paradigms are, however, confronted with the problem that, for the most part, music-syntactic regularities co-occur with acoustic similarity. For example, in a harmonic sequence in *C* major, a *C#* major chord (that does not belong to *C* major) is music-syntactically irregular, but the *C#* major chord is also acoustically less similar to the *C* major context than any other chord belonging to *C* major (because the *C#* major chord consists of tones that do not belong to the *C* major scale). Thus, any experimental effects evoked by such a *C#* major chord cannot simply be attributed to music-syntactic processing. Because such a *C#* major chord is (in its first inversion) the enharmonic equivalent of a Neapolitan sixth chord, it is highly likely that effects elicited by such chords in previous studies e.g., [19], [8]–[10] are not entirely due to music-syntactic processing, but also at least partly due to acoustic deviances that occurred with the presentation of the Neapolitan chords for further details see also [11]. In fact, tonal hierarchies, and music-syntactic regularities of major-minor tonal music are largely grounded on acoustic similarities e.g., [20]. The aim to disentangle the “cognitive” mechanisms (related to music-syntactic processing) from the “sensory” mechanisms (related to the processing of acoustic information) has a certain tradition in music-psychological research (for overviews see, e.g., the special issue of Music Perception 17 (4), 2001), and several experimental paradigms have been suggested to avoid the confound of music-syntactic and acoustic regularity [21]–[23].

One of these paradigms used the homophonic chord sequences presented in Figure 1B [11]. The sequence in the upper panel of Figure 1B ends on a regular tonic chord, the lower sequence ends on an irregular double dominant (DD). Compared to the final tonic, the DD was supposedly acoustically even more similar to the previous four chords: Whereas tonic chords contained two new pitches (in both the top voice and the base voice, see the *e* and the *c* indicated by the arrows in the upper panel of Figure 1B), DDs contained only one new pitch (in the top voice, see arrow in the lower panel of Figure 1B). Acoustic modelling using the IPEM toolbox from Leman et al. [24] confirmed the assumption that the pitch images of the final DDs correlated even higher than those of final tonic chords with the pitch images established by the first four chords (for details see also [24], [11]).

However, in contrast to tonic chords, these DDs introduced a new pitch class (a pitch class is a set of all pitches that are separated by octaves, e.g. the pitch class C consists of the Cs in all octaves, and the C major scale consists of the pitch classes C, D, E, F, G, A, B; that is, a C played in a lower register by a cello has the same pitch class as a C played in a higher register by a violin): For example, in C major a DD introduced the new pitch class F# (indicated by the arrow pointing to the DD in Figure 1B). The DDs thus introduced a pitch that had not been presented either one octave lower or one octave higher in the previous harmonic context. Therefore, the ERP effects elicited by the DDs could still have been driven partly by the occurrence of a new pitch class which was perceptually less similar to the tones occurring in the previous harmonic context (compared to pitches of the final tonic).

For the present study, we composed new chord sequences (shown in Figure 1C) in which the all pitch classes of DDs were also presented in the previous harmonic context. Moreover, in contrast to the sequences shown in Figure 1B (which began with a tonic chord), the new sequences began with a dominant, avoiding that final tonic chords sounded more regular simply because they repeated the first chord function of the sequence. Finally, our new sequences were also composed in a more polyphonic fashion, containing auxiliary notes and passing notes (see 8^th^ notes in Figure 1C), making the sequences sound more natural than the sequences shown in Figure 1B.

**Figure 2:**
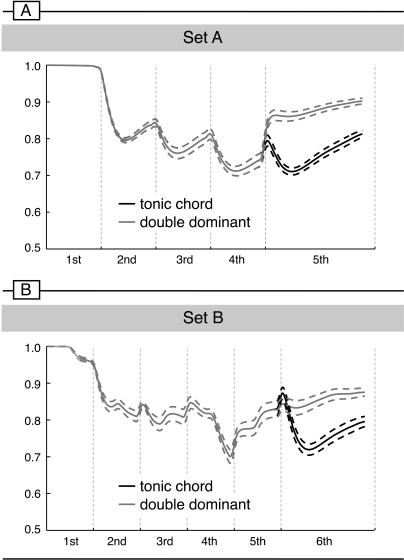
Correlation of local context (pitch image of the current chord) with global context (echoic memory representation as established by previously heard chords), separately for Set A (homophonic sequences, top) and B (polyphonic sequences, bottom). The data show that music-syntactically irregular chord sequence endings (DDs: grey line) were even more congruent with the preceding harmonic context than music-syntactically regular endings (final tonics: black line). For each sequence type, correlations were calculated for all twelve major keys (the line for each sequence type represents the mean correlation, the dashed lines indicate standard error of mean). Auditory modelling was performed using the Contextuality Module of the IPEM-Toolbox (Leman et al., 2005), length of local context integration window was 0.1 sec, global context integration window was 1.5 sec (as suggested by Leman, 2000). The abscissa represents the time line (each chords had a duration of 500 ms, except the last chord which was presented for 1000 ms), the ordinate depicts correlation values. Note that the dip at the beginning of the final tonic in the bottom panel does not represent a statistically significant difference between final tonics and final DDs (t(11) = 0.378, p>.7, t-test for paired samples comparing the mean pitch commonality values of DDs vs. final tonics within a time window from 0 to 240 ms after the onset of the final chord).

That is, in the polyphonic sequences shown in Figure 1C, all pitch classes of DDs occur in the previous harmonic context (i.e., all notes of DDs occurred one or two octaves above or below in the previous context), and DDs repeated even more pitches of the preceding chords than tonic chords did. In addition, the DDs had more pitches in common with the penultimate chord, thus the “sensory dissonance” between final and penultimate chord (of which pitch commonality is the major component) was not greater for DDs than for final tonics. Final DDs were hence acoustically even more similar to the preceding acoustic context than final tonic chords were. Acoustic modelling confirmed the assumption that the pitch images of the final DDs correlated even higher than those of final tonics with the pitch images established by the previous chords (see Figure 2, see Methods for details). Also note that the superposition of intervals was identical for both final tonics and DDs. Because sequences were presented in different keys during the experiment, physically identical chords were music-syntactically regular in one sequence, but irregular in another (for example, the final tonic chord of Figure 1C was a DD of sequences starting in *B-flat* major, and the final DD of Figure 1C was a tonic in sequences starting in *D* major). Therefore, any effect elicited by a DD could not be due to the properties of the chord itself.

Because a previous study has reported that DDs of the sequences depicted in Figure 1B elicit an early right anterior negativity (ERAN) in the ERPs, we expected to replicate this effect in the present study. Moreover, we hypothesized that the DDs of Figure 1C would also elicit an ERAN, despite the fact that they are acoustically even more similar to the preceding context than tonic chords. Note that, hence, mismatch effects elicited by DDs could only be due to their syntactic irregularity, and thus reflect cognitive components of music-syntactic processing. To investigate effects of long-term musical training on the ERAN, we measured two groups of participants (musicians and nonmusicians) with the hypothesis that the ERAN is larger in the group of musicians. This hypothesis was based on two previous studies showing similar training effects [25], [11].

## Methods

### Participants

Data were collected from 12 musicians (mean age: 25.58 years, age range: 23–28, 6 females) and 12 nonmusicians (mean age: 23.42 years, age range: 19–27, 6 females). Musicians had learned at least one musical instrument, with an average formal musical training of *M* = 14.25 years (range: 8–20). Nonmusicians did not have any formal musical training besides normal school education, and they had never learned to play a musical instrument. All participants were right handed (mean of laterality quotient = 93.79%) according to the Edinburgh Handedness Inventory [26], and reported to have normal hearing and no neurological disease. Written informed consent was obtained, the study was approved by the local ethics committee of the University of Leipzig, and conducted in accordance with the Declaration of Helsinki.

### Stimuli

Stimuli consisted of two sets of stimuli, Set A (homophonic sequences of Figure 1B) and Set B (polyphonic sequences of Figure 1C). Set A consisted of two chord sequences, each consisting of five chords (one with a regular ending, one with an irregular ending, Figure 1B). These two sequences were transposed to the twelve major keys, resulting in 24 different sequences (these stimuli had already been used in a previous study, see [11]). The first four chord functions were identical in both sequence types (tonic - subdominant - supertonic - dominant), the final chord of the regular sequence type (upper panel of Figure 1B) was a tonic, the final chord of the irregular sequence type (lower panel of Figure 1B) a double dominant (DD). Using only two sequences transposed to different keys gave us the maximum acoustic control of the musical stimulus (for studies investigating music-syntactic processing with more naturalistic stimuli see, e.g., [27], [28]).

Set B also consisted of two sequences (one with a regular ending, one with an irregular ending, Figure 1C) that were transposed to the twelve major keys, resulting in 24 different sequences. Like in Set A, both sequences differed only with respect to the final chord, the first chords were identical. Sequences began with a dominant upbeat, followed by a tonic, a subdominant, a supertonic, and a dominant. The final chord of the regular sequence type (upper panel of Figure 1C) was a tonic, the final chord of the irregular sequence type (lower panel of Figure 1C) a double dominant (DD, as in Set A). Additionally, eighth notes (auxiliary and passing notes) were introduced in a polyphonic fashion. Presentation time of chords was 500 ms, except for the final chords which lasted 1000 ms followed by a 1000 ms pause.

Sound files of sequences were generated using Cubase SX 2.0 (Steinberg Media Technologies, Hamburg, Germany) with a grand piano sound (Steinberg, The Grand), velocity was identical for all notes. In addition to the sequences only played by a piano sound, we generated sequences with one chord being played by a deviant instrument (bells, VST-sound a1). Such timbre deviants occurred with equal probability at any position of the sequences (they were used only to provide participants with an easy detection task, see below). Across the experiment, each sequence was presented 10 times (two of which contained a chord played by a deviant instrument), resulting in 480 sequences in total. Stimuli of Set A and Set B were presented in blocks, regular and irregular sequences occurred equiprobably (*p* = .5), consecutive sequences always had a different tonal key, and not more than 3 sequences of the same type (regular or irregular) followed each other.

As noted in the Introduction, DDs were in terms of pitch repetition acoustically even more similar to the preceding context than final tonic chords. Thus, DDs should match with the acoustic information stored in the auditory memory traces established by the preceding chords at least as well as final tonics. To test this, we modelled the acoustic congruency of the final chords with the auditory sensory memory traces established by the first chords using the IPEM toolbox [20], [24].

This auditory modelling estimates the pitch images of the echoic memory: Acoustic information decays, but is kept in the echoic memory for a certain time. The aim of the modelling was to determine the correlation of the pitch image of a final chord with the pitch image of preceding chords stored in the echoic memory. The results of the modelling are shown in Figure 2A and B (echo of local images: 0.1 s, echo of global image: 1.5 s, see [20], note that these values indicate half decay values, and that -particularly due to the use of the 1.5 s gliding window - information of all preceding chords affects the correlations between the last chord and the preceding chords). In both sets of sequences (Set A and Set B), the pitch images of the final DDs correlated even higher than those of final tonic chords with the pitch images established by the preceding chords.

### Procedure

Sequences of Set A (homophonic, Figure 1B) and Set B (polyphonic, Figure 1C) were presented in different blocks, counterbalanced across subjects. Each block was further subdivided into two sub-blocks. In the first sub-block, participants looked at a fixation cross while listening to the stimuli; in the second sub-block, they watched a silent movie (without subtitles, reduced to 1/4th of its original size to reduce eye movement artefacts). The duration of the experiment was approximately 60 minutes.

Participants sat in a comfortable chair in a sound proof cabin. Stimuli were presented via loudspeakers at a comfortable volume using *Presentation 0.52* software. Participants were not informed about the regular and irregular sequence endings. Instead, they were informed about the deviant instruments, and asked to respond to them by pressing a button. This task has already been used in a number of previous studies e.g., [8], [29], [10], [30], [11] and allowed us to control that participants attended to the auditory stimulus, without requiring them to detect the irregular chords (such a conscious detection elicits N2b and P3b potentials, the N2b overlapping with the ERAN, and the P3 overlapping with the N5, thus obscuring the brain responses related to the music-syntactic analysis of the chords, see e.g. [8]). Furthermore, it is worth noting that entertaining the subjects with a (silent) movie improves the quality of the EEG data because less strain is put onto the subjects, particularly during longer recording sessions (such strain usually produces noise in the EEG data due to muscle tension and excessive eye blinking). Hence, more trials can be recorded (and included in the analysis), increasing the signal-to-noise ratio of the data. Note that the timbre detection task was not used to differentiate between groups or stimulus sets.

### Data Recording and Analysis

The EEG was recorded with 32 Ag/AgCl cap-mounted electrodes (Electrocap International) according to the extended 10–20 system (FP1/2, AF7/8, AF3/4, AFZ, F7/8, F3/4, FZ, FT7/8, FC3/4, T7/8, C3/4, CZ, CP5/6, P7/8, P3/4, PZ, O1/2). The left mastoid (M1) served as reference; additional electrodes were placed on the nose-tip and the right mastoid (M2). The ground electrode was located on the sternum. To monitor eye movements and blinks, horizontal and vertical electrooculograms (EOG) were bipolarly recorded from electrodes placed on the outer canthus of each eye (horizontal EOG), as well as above and below the right eye (vertical EOG). Impedances were kept below 5 k-Ohm. Signals were amplified with two synchronised PORTI-32/MREFA amplifiers (Twente Medical Systems International BV) and digitised with a sampling rate of 250 Hz.

After the measurement, data were re-referenced to the mean of both mastoids, and filtered using a 0.5–20-Hz bandpass filter (fir, 1001 points, −6 dB/octave, hamming window). For artefact reduction, EEG data were rejected whenever the *SD* of the signal recorded at any electrode exceeded 25 microvolts within a 200-ms or 800-ms gliding window. Additionally, trials with typical eye blinks were marked and corrected by applying electrooculogram correction (xeog, EEP software, ANT, Netherlands). Finally, ERPs were calculated separately for the regular and irregular final chords of each set using a 200-ms prestimulus baseline and a 1000-ms poststimulus window. Sequences containing deviant instruments were excluded from further analysis (because they were only employed to devise a task for the subjects, see also above).

**Figure 3:**
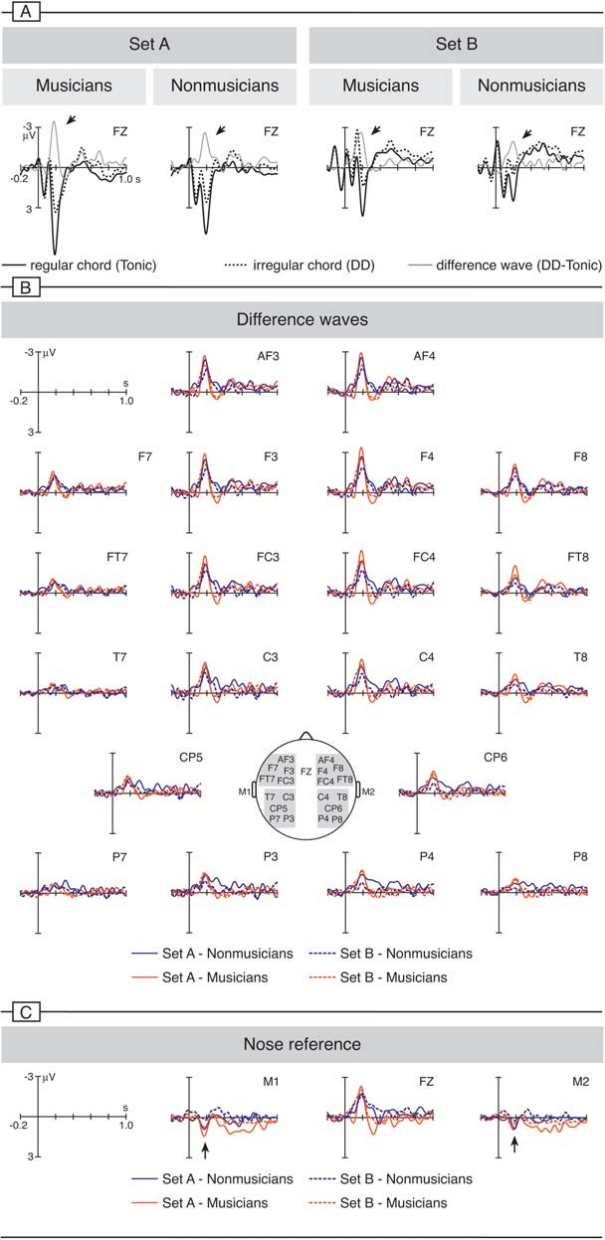
Grand-average ERP waveforms. A: ERPs elicited by the final chords at a frontal electrode (Fz, referenced to the algebraic mean of both mastoid electrodes). The thick solid line indicates potentials elicited by regular (tonic) chords, the dotted line responses to irregular chords (double dominants). The thin solid line represents the difference wave (regular subtracted from irregular chords). DDs elicited in both sets (A and B), and in both groups (musicians and nonmusicians) an early right anterior negativity (ERAN, see arrows). As can best be seen in the difference waves of B (regular subtracted from irregular chords, referenced to both mastoid electrodes), the ERAN was larger in Set A (solid lines) than in Set B (dashed lines), and larger in musicians (red lines) than in nonmusicians (blue lines). The inset depicts head positions of the electrodes shown in A and B, regions of interest used for statistical analyses are shaded in grey. C: When referenced to the nose electrode, the ERAN inverted polarity at mastoid leads (M1, M2, the polarity inversion is indicated by the arrows).

For statistical analysis, mean amplitude values within a time window from 160 to 200 ms (centred around the ERAN peak) were calculated for 4 Regions of Interest (ROIs; see also inset of Figure 3B): left anterior (AF3, F7, F3, FT7, FC3), right anterior (AF4, F8, F4, FT8, FC4), left posterior (T7, C3, CP5, P7, P3), and right posterior (T8, C4, CP6, P8, P4). An ANOVA with repeated measures factors Chord (regular [Tonic] vs. irregular [DD]), Hemisphere (left vs. right), Anterior-Posterior (anterior vs. posterior), Set (A vs. B), Visual Stimulus (fixation cross vs. movie), and the group factor Expertise (musicians vs. nonmusicians) did not indicate a main effect of Visual Stimulus, and no interaction involving Visual Stimulus and Chord. Hence, data of the blocks with fixation cross and silent movie were pooled separately for each set, resulting in a 5-way ANOVA with factors Chord, Hemisphere, Anterior-Posterior, Set, and Expertise. Whenever an interaction was observed at a significance level of *p* = .05, subsequent analyses were conducted by splitting up the general linear model. The same analysis was conducted for a later time window from 450 to 700 ms covering the N500.

Behavioural data (hit rates of timbre deviants) were analyzed by an ANOVA with repeated measures factors Set (A vs. B), and the group factor Expertise (musicians vs. nonmusicians).

## Results

Participants detected on average 99.48% of the deviant instruments, reflecting that that this task was easy (as intended), and showing that individuals attended to the musical stimulus (hit rates did not significantly differ between Sets, *p*>.08, or between groups, *p*>.1).

Figure 3A shows the electric brain responses to harmonically regular and irregular sequence-endings, separately for the two sets of sequences, and separately for nonmusicians and musicians. In both sets of sequences, irregular DDs elicited an ERAN that was maximal over fronto-midline electrodes, and that had slightly larger amplitude values over right than over left-hemisphere electrode sites (see difference waves of Figure 3B). At frontal sites, the ERAN was larger when elicited by the (homophonic) sequences of Set A than when elicited by the (polyphonic) sequences of Set B, and the ERAN tended to be larger in musicians than in nonmusicians. With nose reference, the ERAN inverted polarity at mastoid leads at around 200 ms (Figure 3C), indicating that this ERP effect is not an N2b (the N2b has a central maximum, is not lateralized, and does not invert polarity at mastoid sites [31], [32]).

A global ANOVA with the repeated measures factors Chord (regular [Tonic] vs. irregular [DD]), Set (A vs. B), Hemisphere (left vs. right), Anterior-Posterior (anterior vs. posterior), and the between subjects factor Expertise (musicians vs. nonmusicians) revealed a main effect of Chord (*p*<.0001, reflecting that irregular chords elicited an ERAN), an interaction of Chord×Hemisphere (*p*<.007, reflecting that the ERAN was right-lateralized), and an interaction of Chord×Anterior-Posterior (*p*<.0001, reflecting that the ERAN was larger over frontal than over parietal sites; details of the ANOVA are provided in Table 1). There was a significant three-way interaction of Chord×Set×Anterior-Posterior (*p*<.033, reflecting that the ERAN amplitude differed between both sets of sequences at anterior leads), and a follow-up ANOVA with factors Chord, Hemisphere, Set, and Expertise computed separately for anterior ROIs yielded a significant interaction of Chord×Set (*F*(1,22) = 4.79, *p*<.039), indicating that DDs of Set A elicited a larger ERAN than DDs of Set B at anterior electrode sites.

**Table 1:** Summary of global ANOVA for the ERAN time window (160–200 ms) with factors Chord (regular, irregular), Hemisphere (left, right), Anterior-Posterior (anterior, posterior), Set (A, B), and Expertise (nonmusicians, musicians).

	*F*_1, 22_ and *P*-values
Chord	**148.48, 0.0001**
Hem.	9.38, 0.006
AntPost	15.16, 0.001
Set	113.37, 0.0001
Set×Exp.	4.55, 0.045
Chord×Hem.	**8.88, 0.007**
Chord×AntPost	**29.87, 0.0001**
Hem.×AntPost	6.66, 0.018
Hem.×Set	4.33, 0.050
AntPost×Set	39.75, 0.0001
Chord×Hem.×Exp.	**3.32, 0.082**
Chord×Hem.×Set	**3.53, 0.074**
Chord×AntPost×Set	**5.22, 0.033**
Hem.×AntPost×Set	7.23, 0.014

Only main effects and interactions with p<.1 are reported. Bold font indicates main effects and interactions involving the factor Chord.

The interaction of Chord×Expertise missed the level of significance in the global ANOVA (*F*(1,22) = 2.88, *p* = .104), but is marginally significant when tested one-sided (*p*<.06) according to the hypothesis that the ERAN is larger in musicians than in nonmusicians (see Introduction).

In both stimulus sets, the ERAN was followed by a late anterior negativity that was maximal at around 500 ms, the N5 [8]. The N5 was slightly right-lateralized, and did not differ between musicians and nonmusicians. A global ANOVA (analogous to the one carried out for the ERAN) with a time window from 450 to 700 ms revealed a main effect of Chord (F(1,22) = 21.54, p<.001), an interaction of Chord×Anterior-Posterior (F(1,22) = 7.28, p<.014), and an interaction of Chord×Hemisphere (F(1,22) = 5.67, p<.027). Moreover, an interaction of Chord×Set was observed (F(1,22) = 4.32, p<.050, reflecting that the N5 was slightly larger when elicited by the stimuli of Set A than by those of Set B). Follow-up ANOVAs with factors Chord, Hemisphere, Anterior-Posterior, and Expertise, calculated separately for each set of stimuli, showed that stimuli of both sets elicited a significant N5 (main effect of Chord for Set A: F(1,22) = 19.58, p<.001; and Set B: F(1,22) = 5.88, p<.025).

## Discussion

Results showed that both sets of sequences (Figure 1B and C) elicit an ERAN. The elicitation of an ERAN of the homophonic sequences of Set A replicates results of a previous study [11]. However, DDs of these sequences contained a new pitch class (this note was not contained in any of the previous chords, see the *F#* indicated by the arrow of the DD in Figure 1B), in contrast to final tonic chords, of which all pitches had been presented either one octave lower or one octave higher in the previous harmonic context. That is, the ERP effects elicited by these DDs could still partly be driven by the occurrence of a new pitch class which was perceptually less similar to the pitches occurring in the previous harmonic context than pitches of the final tonic.

To avoid this possible confound, we also used sequences in which notes of DDs already had been presented in the previous harmonic context (Set B, bottom panel of Figure 1C). Results showed that these DDs also elicited an ERAN, demonstrating that the generation of this ERP component is not dependent on the occurrence of new pitch classes (i.e., new notes), and indicating that the ERAN effect is largely due to music-syntactic processing (and not due to pitch repetition effects). It is still true that the pitch class of the new note introduced by DDs occurred only once in the previous context, whereas the pitch class of the top voice of final tonic chords occurred twice, but it is highly unlikely that this accounts for the ERAN effect, particularly because the ERAN can even be elicited when irregular chords do not introduce any new pitch class [33]. Future studies could manipulate the number of previous presentations of the tones included in the final chords in different experimental blocks.

The ERAN amplitude was larger when elicited by sequences of Set A (homophonic) than by those of Set B (polyphonic), for which several reasons might account: (1) The occurrence of a new (out-of-key) note in the DDs of the homophonic sequences made DDs slightly more unexpected than DDs of the polyphonic sequences. (2) The difference in sequence length (the homophonic sequences were five chords long, the polyphonic sequences consisted of six chords) might have led to an interaction between music-syntactic processing and working memory operations. (3) Polyphonic sequences did not begin with a tonic chord, perhaps making the extraction of the tonal centre more difficult than for homophonic sequences. Theses issues could be specified in future studies.

The ERAN tended to be larger in musicians than in nonmusicians (although this group difference was statistically only marginally significant), which is in line with some previous studies that reported larger ERAN amplitude values for musicians [25], and for amateur musicians [11] compared to nonmusicians. In the latter study [11], the difference between groups was just above the threshold of statistical significance, and in a recent study from Koelsch & Jentschke [33], the difference in ERAN amplitude between amateur musicians and nonmusicians (with amateur musicians showing larger amplitude values) did not reach statistical significance. The combined results suggest that the ERAN is modulated by long-term musical training, that these training effects are small, but that they are reliable and consistent across studies. The ERAN is presumably larger in musically trained individuals because they have more specific representations of music-syntactic regularities and are, thus, more sensitive for violations of these regularities.

The ERAN was followed by an N5 that was maximal around 500–550 ms and had a right-lateralized scalp distribution. The N5 is taken to reflect processes of harmonic integration [8], [13]: The first chords of the sequences build up a harmonic context towards the end of the sequence. Regular final chords (tonics) can easily be integrated into the established musical context, whereas irregular chords (DDs) require a larger amount of harmonic integration (because they do not easily fit into the harmonic fabric established by the first chords). The processes of harmonic integration appear to resemble processes of semantic integration during the perception of language (indexed by the N400; e.g., [34]), and might at least partly reflect processing of musical meaning (irregular chord functions, and deceptive cadences, are prominent elements of major-minor tonal music that are used by composers as a means of expression [35], [13]). The exact relation between N5 and processing of musical meaning, however, remains to be specified.

### Conclusions

The present study used polyphonic chord sequences with music-syntactically regular and irregular endings, in which sensory factors such as pitch repetition, and pitch commonality with the preceding chord (which is the major component of “sensory dissonance”) could hardly contribute to the elicitation of ERP effects of irregular chords. Irregular chords nevertheless evoked an ERAN, showing that the ERAN effect is not dependent on the occurrence of sensory deviance, and that the ERAN effect elicited in the present study by the DDs of polyphonic sequences reflects for the most part cognitive music-syntactic processing. Hence, the sequences presented in this study are particularly suited to investigate music-syntactic processing, its development, its impact on emotion, and its relation to language-syntactic processing.
